# Unravelling the small number bias: the role of pseudoneglect and frequency of use in random number generation

**DOI:** 10.1007/s00426-025-02101-8

**Published:** 2025-03-25

**Authors:** Serena Mingolo, Valter Prpic, Alberto Mariconda, Tiziano Agostini, Mauro Murgia

**Affiliations:** 1 Department of Physics, University of Calabria, Rende, Italy; 2 Department of Life Sciences, University of Trieste, Trieste, Italy; 3 Department of Theoretical and Applied Sciences, eCampus University, Novedrate, Italy; 4 Department of Philosophy, University of Bologna, Bologna, Italy

## Abstract

When asked to produce random numbers individuals generate more small numbers than large ones, a phenomenon known as “Small Number Bias” (SNB; Loetscher & Brugger, 2007). This bias has been associated with a spatial preference known as “pseudoneglect,” where attention is biased towards the left side of the mental number line during numerical processing (Loetscher & Brugger, 2009). Another potential explanation for SNB is the higher frequency of use of small compared to large numbers in daily life (Dehaene & Mehler, 1992). This study aims to determine which of these two explanations better accounts for SNB. Participants were asked to generate random numbers from 1 to 12 while viewing either a regular or an inverted clockface. On a regular clockface smaller numbers are on the right, whereas on an inverted clockface they are on the left. Both theories predict SNB for the inverted clockface. However, for the regular clockface, frequency of use would predict SNB, while pseudoneglect would predict a bias towards larger numbers. Results showed SNB in the inverted clockface condition, but no bias in the regular clockface condition. These findings suggest that SNB arises when pseudoneglect and frequency of use align but is absent when they conflict. Overall, the results indicate that both pseudoneglect and frequency of use contribute to SNB in some degrees.

## Introduction

Numerical cognition is a crucial determinant of human life, involved in activities ranging from simple arithmetic to complex decision-making. Understanding how we process numbers can not only explain how we count and perform mathematics but can also shed light on broader cognitive mechanisms. Although the concept of number is formally taught in school, studies have shown that humans are capable to operate with quantities even before this knowledge is acquired, at a preverbal stage (Xu & Spelke, 2000; Xu et al., 2005). This ability suggests that our brains are inherently equipped to handle numerical information. Numbers are deeply embedded in our cognitive system, with small quantities holding a special place. The term “subitizing”, (Kaufman et al., 1949), refers to the rapid and accurate enumeration of quantities less than four. Due to subitizing, we can immediately grasp the numerosity of small sets of items without serially counting them, whether these items are presented visually or in other perceptual modalities (Katzin et al., 2019).

Our perception of numbers is closely linked with our perception of space (for a review see Wood et al., 2008). Since the early reports of people visualizing numbers in recurrent spatial layouts (Galton, 1880), the most popular spatial-numerical representation has been the “mental number line” (MNL; Restle, 1970). This MNL, extending from left to right in imagined space, accounts for common phenomena in numerical cognition, such as the SNARC effect (Dehaene et al., 1993). The SNARC effect emerges in tasks where numbers are categorized based on criteria like parity or magnitude using lateralized responses. The response patterns to these tasks show that smaller numbers are typically associated with the left side of space and larger numbers with the right, according to the MNL. This effect, which has been observed not only with numbers, but also with different numerical formats (Nuerk et al., 2005; Prpic et al., 2023), is related to spatial compatibility, which predicts fast and accurate processing when response effectors are placed on the same side as the stimuli (Fitts & Seeger, 1953).

One intriguing phenomenon related to our representation of numbers which might as well be related to space is the Small Number Bias (SNB), which is a systematic preference for small numbers in healthy subjects (Loetscher & Brugger, 2007). This bias often emerges in a task called “random number generation” (RNG), which requires to enumerate random numbers within a specified range. Instructions for this task are often exemplified with the “mental urn” metaphor: participants are instructed to orally enumerate numbers as drawing them from an imaginary “urn” in their brain (Dehaene, 2011). Variations of this task incorporating spatial variables have revealed further spatial biases in number production. For instance, SNB is noted when participants are instructed to turn their heads left and is more intense if they visualize a ruler while doing so (Loetscher et al., 2008a, b). Additionally, spontaneous eye movements can predict the magnitude of the number a participant is about to name, with downward/leftward eye movements increasing the likelihood of a smaller number and upward/rightward eye movements increasing the likelihood of a larger number (Loetscher et al., 2010).

Despite its significance, the mechanisms behind SNB remain debated. One theory, known as “pseudoneglect” (Bowers & Heilman, 1980; Brooks et al., 2014; Jewell & McCourt, 2000) suggests that people’s attention is automatically biased toward the left portion of space, while the right space is partly neglected. This left-side preference is believed to stem from an evolutionary adaptation favouring left-to-right attentional scanning (Rugani et al., 2015; Dissegna et al., 2023). The right hemisphere’s asymmetrical specialization in processing visuospatial and numerical information likely accounts for this tendency (Rogers et al., 2013). Thus, smaller numbers would be more salient due to their spatial position on the left side of the MNL. The influence of spatial attention on performance in various number tasks has been demonstrated not only in neglect patients (Vuilleumier et al., 2004; Zorzi et al., 2002, 2006), but also in healthy individuals (Fischer, 2001; Fischer et al., 2003). This bias leads healthy individuals to preferentially focus on the left side of the imagined number space when processing numbers (Loetscher & Brugger, 2009). For example, in bisection of visually presented horizontal lines, participants often misplace the midpoint to the left of its true position (Jewell & McCourt, 2000). Similarly, when “bisecting” an auditorily presented numerical interval, participants tend to misplace the midpoint toward smaller values, i.e., to the left of the MNL (Brugger et al., 2010).

Another possible explanation for SNB is that small numbers are overrepresented because they are used more frequently than large ones in everyday life (Dehaene & Mehler, 1992). This straightforward explanation suggests that our brains are more receptive to smaller numbers due to their common verbal usage, resulting in a more accurate and detailed representation of these numbers on the MNL. This explanation offers valuable insights into the nature of SNB and deserves further investigation. Importantly, this account does not necessarily conflict with the pseudoneglect hypothesis; the accounts could co-exist and contribute to SNB in different ways. However, they have never been directly compared in empirical studies. Investigating these hypotheses side by side could provide a clearer understanding of SNB and of its visuospatial/verbal nature, potentially revealing which explanation holds more weight under specific conditions. Empirical evidence that directly tests both hypotheses could help determine the relative contributions of attentional biases and frequency of use in the manifestation of SNB, offering a more comprehensive picture of the underlying mechanisms.

**Fig. 1:**
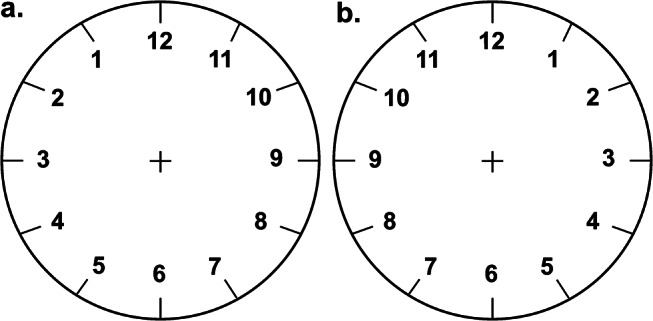
The outlines of a regular clockface (Panel **a**) and that of a “reversed clockface” (Panel **b**) were presented to each participant during random number generation task. The order of presentation of the two pictures was counterbalanced among participants

The general objective of the present study is to investigate the mechanisms underlying the SNB. Specifically, the study aims to evaluate if SNB is better explained by pseudoneglect, which biases attention towards the left side of the space, or frequency of use, which reflects the more common verbal usage of smaller numbers compared to larger numbers. This objective will be achieved by comparing these two possible explanations in a random number generation task. Importantly, the task will be performed with two experimentally manipulated displays of numbers: a regular clockface (Fig. 1a) and a reversed clockface (Fig. 1b). This methodological choice is guided by previous studies that have used clockface displays to explore basic processes like numerical recognition and retention (e.g., the visuospatial bootstrapping study by Mallik et al., 2022) and to investigate cognitive impairments caused by various pathologies (e.g., Hazan et al., 2018, on the history of the clock drawing task), such as hemispatial neglect (Rossetti et al., 2011) and dementia (Aprahamian et al., 2009).

The rationale for using a clockface display to investigate spatial and numerical biases lies in its familiar structure. This peculiar display of numbers is widely recognized, often used in everyday life and familiar to people across various cultures and age groups. However, it differs significantly from the mental number line (MNL), where smaller numbers (e.g., 1–6) appear on the left and larger numbers (e.g., 7–12) appear on the right. In a clockface display, numbers are represented in the opposite spatial positions: smaller numbers on the right and larger numbers on the left (Fig. 1a). This inversion makes the clockface an ideal tool for examining how spatial biases can affect numerical processing.

In a seminal study, Bächtold and colleagues (1998) asked participants to judge the magnitude of numbers presented within a ruler – reflecting the MNL – or a clockface – where the positions of small and large numbers are flipped. In the clockface condition, results revealed a reversal of the commonly observed SNARC effect (Dehaene et al., 1993), which typically determines faster responses to small numbers with the left effector and to large numbers with the right effector. Conversely, participants responded faster to small numbers with the right effector and to large numbers with the left. Later studies indicated that a similar alteration of the SNARC effect only occurs if task instructions explicitly require processing numbers within the context of an alternative numerical display (Mingolo et al., 2021).

Recently, SNB was observed in an RNG task performed on a regular clock (Mingolo et al., 2024). In this case, the clockface was used to see to what extent a reversed MNL could affect a common spatial numerical bias as the SNB. However, the clock context was only presented at the beginning of the task. Thus, even though participants were specifically required to visualize it during RNG, it could not be ascertained that the participants did so. Notably, the level of salience of the context in this kind of task is defined as “low”, and thus its influence on participants’ performance is relatively weak. Indeed, a reliable SNB was observed, showing that the clockface configuration did not affect the bias.

The present study focuses on participants’ performance in generating random numbers ranging from 1 to 12 while numbers are presented within either a regular clockface (Fig. 1a) or an inverted one (Fig. 1b). This manipulation allows to distinguish between the contributions of pseudoneglect and frequency of use to the SNB. If random number generation is influenced by the visually presented numerical display, two predictions can be made based on the theoretical explanations:

- Pseudoneglect predicts a preference for drawing numbers from the left side of the clock. This would be reflected by a tendency to produce large numbers in the regular clockface condition and small numbers in the reversed clockface condition.
- Frequency of use predicts a preference for drawing small numbers, independent of the clock side. This would be reflected by a tendency to produce small numbers in both the regular and reversed clockface conditions.

Thus, if participants prefer small numbers in both conditions, this will support the frequency of use account. Differently, if participants prefer more small numbers in the reversed condition and more large numbers in the regular condition, this will support the pseudoneglect account.

## Methods

### Participants

Seventy-three students (forty-six females and twenty-seven males) were recruited for the study (mean age = 22.46 years, standard deviation = 2.78 years). The sample size was determined by means of the software GPower. The suggested sample size of 71 participants was obtained using the following parameters: power = 0.80, α = 0.05, Cohen’s *d* = 0.30. Previous similar studies reported effect sizes for SNB in the range of medium to large (e.g., *d* = 0.44–0.68; see Mingolo et al., 2024 and Winter & Matlock, 2013); the present power analysis aims to detect small/medium effects as well. All participants had normal or corrected vision and confirmed that their psychophysiological state was not affected by alcohol consumption or insufficient sleep from the previous night (Murgia et al., 2020). They had no prior knowledge of the research aims to ensure unbiased results. Their participation was compensated with academic credits. Prior to data collection, each participant provided written informed consent to take part to the experiment. The study adhered to ethical standards outlined in the Declaration of Helsinki and gained approval from the University of Trieste Ethics Committee.

### Apparatus and stimuli

The experiment was run with an HP PC with Intel Core i7 11th generation (RAM: 16 Gb). The visual stimuli were presented on a 32-in. MSI monitor (Optix Mag 322CR, 180 hz). The auditory stimulus (i.e., an auditory track reproducing the sound of a metronome synchronized at the frequency of 1 Hz, 60 bpm) was presented via a set of Sony MDR-XB950/B headphones. The visual stimuli (i.e., two clockface-like displays) employed in the experiment consisted in two pictures. One picture (Fig. 1a) depicted the outline of a regular clockface, with numbers ranging 1–12 arranged in clockwise order; namely, small numbers (i.e., 1–5) are placed on the right side of the figure, while large numbers (i.e., 7–11) are placed on the left side. The other picture (Fig. 1b) depicted the same outline, but in this case the numbers (1–12) are arranged in the opposite way compared to the first one. Namely, small numbers (i.e., 1–5) are placed on the left side of the figure, while large numbers (i.e., 7–11) are placed on the right side.

### Procedure

The experiment was conducted in a quiet room with dim lighting. Participants wore headphones and sat approximately 60 centimetres from a monitor, with their bodies aligned to the monitor’s midline. After a brief explanation from the experimenter about the experiment’s structure and duration (approximately 5 min), participants were given written instructions displayed on the screen. The experiment consisted of two blocks, each comprising a practice session – whose data were excluded from data analysis – to familiarize with the task, and an experimental session.

Participants performed a task named Random Number Generation (RNG). They were instructed to randomly name a number – among those appearing on the screen – at each beat of a metronome (60 bpm). They were informed that a picture containing numbers would be displayed on the screen, and that only those numbers could be used to perform the task. The numbers displayed in the picture ranged from 1 to 12 and were either depicted as in a regular clockface (Fig. 1a) or as in a reversed clockface (Fig. 1b). The same picture was displayed throughout the whole block; the order of presentation of the two pictures was counterbalanced among participants (i.e., half of the participants saw Fig. 1a first, and the other half saw Fig. 1b first). Participants were instructed to focus their attention on the picture for the entire duration of the session.

### Data analysis

The study followed a within subject design, with participants performing both conditions: one in which the picture presented was a regular clockface, and one in which the picture presented was a reversed clockface. The independent variables were Condition (Regular vs. Reversed) and Number (1, 2, 3, 4, 5, 6, 7, 8, 9, 10, 11, 12), and the main dependent variable was the frequency of occurrence of the numbers.

To explore the pattern of numbers’ production in RNG task, initially a 2 × 12 (Condition x Number) repeated measures ANOVA was performed on all frequencies. Then, a one-sample t test was run to determine if any number was produced with higher/lower frequency than chance level (Table 1). This was achieved by comparing frequencies of occurrence of each number in each condition against the expected frequency of producing a random number in this task (i.e., 5, calculated through the following formula: [1/12] * 60). Then, a paired-sample *t* test was run on the frequencies of occurrence of each number to determine if any number was produced more frequently in any of the two conditions (Table 1).

To inspect the overall presence of SNB, independently from conditions, a paired sample *t* test compared the frequency of occurrence of small numbers against large numbers from both conditions. Then, the same comparisons were run separately for each condition considering different sets of numbers, in order to determine if any of the two conditions (Regular vs. Reversed) presented the SNB. Finally, the differences between the frequencies of small and large numbers from the two conditions were compared among each other through a paired sample *t* test.

**Fig. 2:**
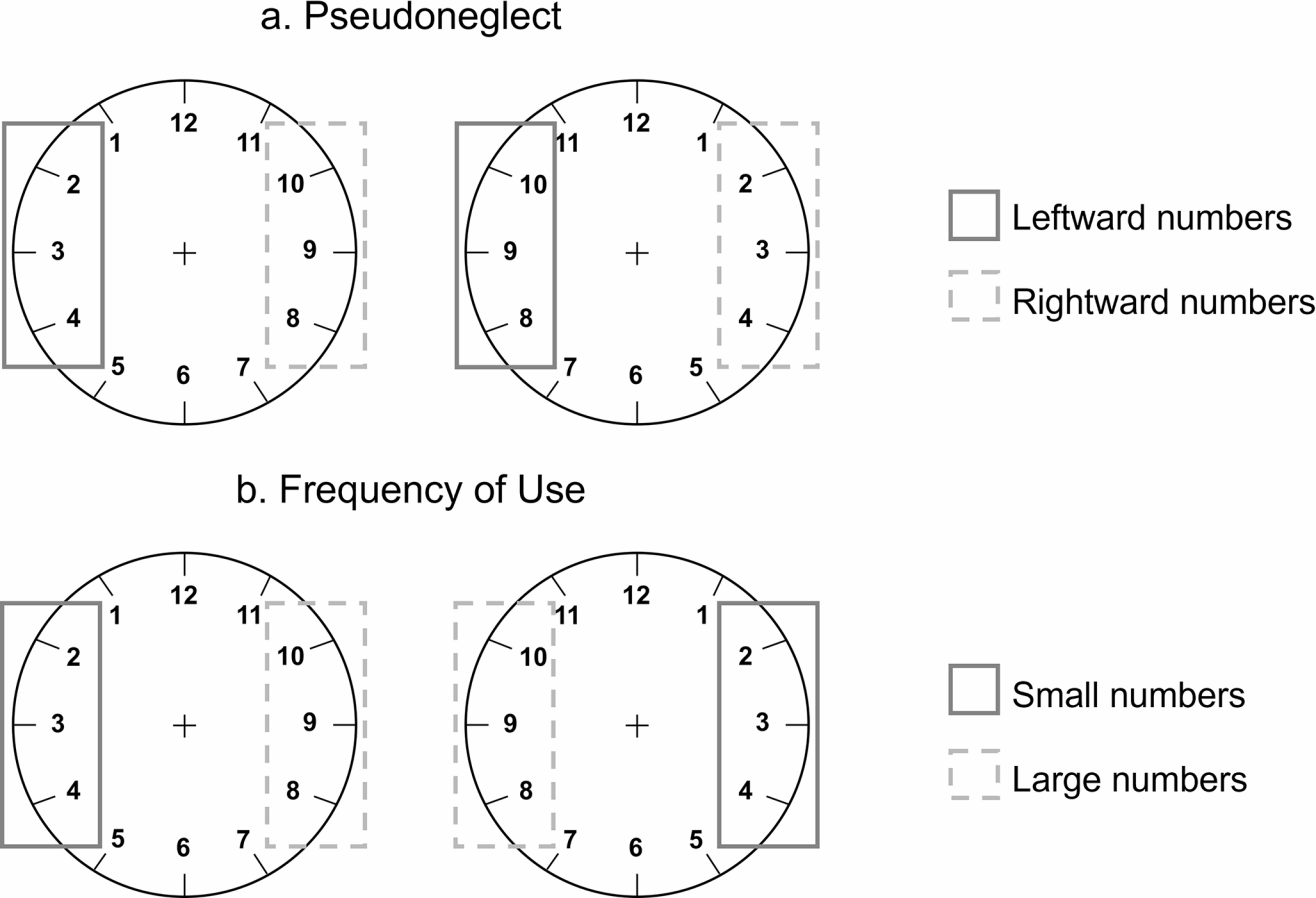
The figure reflects the two accounts objective of study: pseudoneglect (panel **a**) and frequency of use (panel **b**). The numbers highlighted in the figure were used to run a set of two paired sample t tests in order to reveal the contribution of both accounts to SNB

Finally, to determine which account between pseudoneglect and frequency of use better explains the pattern of number production observed in participants’ performance, two variables were computed and analysed. First, to reflect pseudoneglect, the average frequency of production of the most leftward and most rightward numbers from the two configurations were computed and compared in a paired sample *t* test (i.e., leftward: sum of numbers 8, 9 and 10 in the regular clockface and numbers 2, 3, and 4 in the reversed clockface; rightward: numbers 2, 3, and 4 in the regular clockface and numbers 8, 9 and 10 in the reversed clockface; Fig. 2a). Second, to reflect frequency of use, the average frequency of production of the smallest and largest numbers from the two configurations were computed and compared in a paired sample *t* test (i.e., smallest: numbers 2, 3, and 4 in the regular and reversed clockface; largest: numbers 8, 9 and 10 in the regular and reversed clockface; Fig. 2b). Depending on these results, it could be determined if some numbers had been produced more frequently due to their spatial position (pseudoneglect) or their magnitude (frequency of use).

## Results

The 2 × 12 (Condition x Number) repeated measures ANOVA that was run to explore the frequencies of production of numbers revealed a significant main effect of the variable Number [*F*(11, 792) = 5.36; *p* <.001; η_p_^2^ = 0.069], indicating that some numbers were produced more frequently than others. No significant main effect for the variable Condition emerged, due to the task instructions (which required generating a fixed amount of numbers in both conditions). The interaction resulted to be significant [*F*(11, 792) = 1.94; *p* =.031; η_p_^2^ = 0.026], indicating that some numbers were produced more frequently in one condition compared to the other. Table 1 reports the average frequencies for each number in each condition, as well as the significant deviations from the expected value.

**Table 1:** Mean and standard deviations of the frequencies for each number in each condition. Frequencies significantly deviating from the expected value (i.e., 5) are marked with asterisks (*p <.05; **p <.001). Differences between conditions were also tested, revealing a significant difference only for number 11 (*p* =.026)

	Numbers											
	1	2	3	4	5	6	7	8	9	10	11	12
Regular	4.97	4.85	5.32	5.22	5.03	4.44**	5.26	4.88	5.22	5.32*	4.33**	4.82
	(1.46)	(1.50)	(1.71)	(1.54)	(1.48)	(1.35)	(1.51)	(1.54)	(1.33)	(1.46)	(1.42)	(1.35)
Reversed	4.71*	5.14	5.55**	5.18	5.15	4.59*	4.96	4.81	5.38*	4.95	4.75	4.56
	(1.35)	(1.28)	(1.37)	(1.26)	(1.42)	(1.20)	(1.49)	(1.41)	(1.52)	(1.57)	(1.35)	(1.44)

**Fig. 3:**
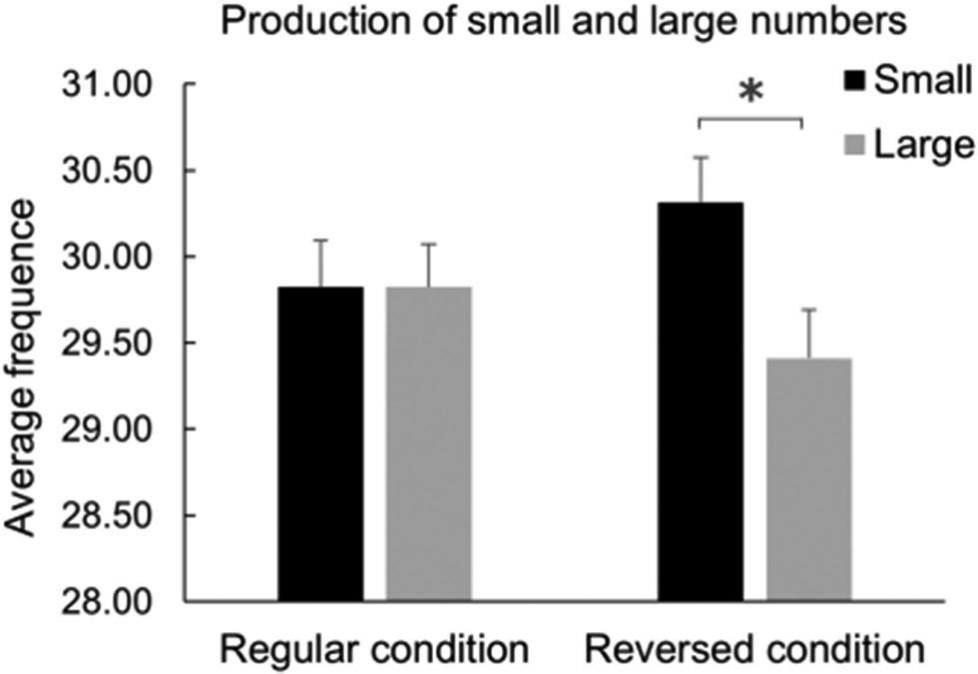
Mean frequencies of small vs. large numbers generated in the Regular and Reversed conditions. A significant difference was found in the Reversed condition. Errors bars indicate the standard error of the mean

The one-tailed paired sample *t* test that compared the frequency of occurrence of small numbers (i.e., 1–2 – 3–4 – 5–6) against large numbers (i.e., 7–8 – 9–10 – 11–12) from both conditions revealed a tendency toward a significant difference [*t*(72) = 1.59, *p* =.057, *d* = 0.187], reflecting an overall tendency towards SNB in the RNG task, independently from conditions. Two one-tailed paired sample *t* tests were then run to compare the frequency of occurrence of small numbers against large numbers, separately for each condition. Considering the range of small (i.e., 1–2 – 3–4 – 5–6) vs. large (7–8 – 9–10 – 11–12) numbers, in the Regular condition no significant difference emerged [Δ = 0, *t*(72) = 0.00, *p* =.500, *d* = 0.000]; in the Reversed condition, a significant difference in the direction of the SNB emerged [Δ = 66, *t*(72) = 1.73, *p* =.044, *d* = 0.202]; see Fig. 3. The one-tailed paired-sample *t* test that compared such differences did not reveal a significant difference between the two conditions [*t*(72) = 1.09, *p* =.139, *d* = 0.128].

Notably, a further set of one-tailed one-sample *t* tests was then run on the same variables (i.e., small vs. large numbers) considering different ranges of numbers (i.e., those spatially located on the left/right of the configuration and those at the extremes). The results (Table 2) further indicate the presence of SNB only in the reversed condition.

**Table 2:** Summary of results from one-tailed paired sample t tests comparing small vs. large numbers in the regular and reversed conditions

Numerical range	Condition	Students t	df	*p* value	ΔMean	Cohen’s d
1, 2, 3, 4, 5 vs. 7, 8, 9, 10, 11	Regular	0.81	72	0.21	0.38	0.09
1, 2, 3, 4, 5 vs. 7, 8, 9, 10, 11	Reversed	1.91	72	0.03	0.88	0.22
2, 3, 4 vs. 8, 9, 10	Regular	0.06	72	0.52	0.02	0.01
2, 3, 4 vs. 8, 9, 10	Reversed	1.87	72	0.03	0.72	0.22

Finally, a last set of one-tailed paired sample *t* tests was performed, with the aim of understanding which account between pseudoneglect and frequency of use could best explain the pattern of observed results. These tests did not detect a significant difference between the production of leftward vs. rightward numbers [*t*(72) = 1.08, *p* =.143, *d* = 0.126], but revealed a difference that tends towards significance between the production of small vs. large numbers [*t*(72) = 1.60, *p* =.057, *d* = 0.187].

**Fig. 4:**
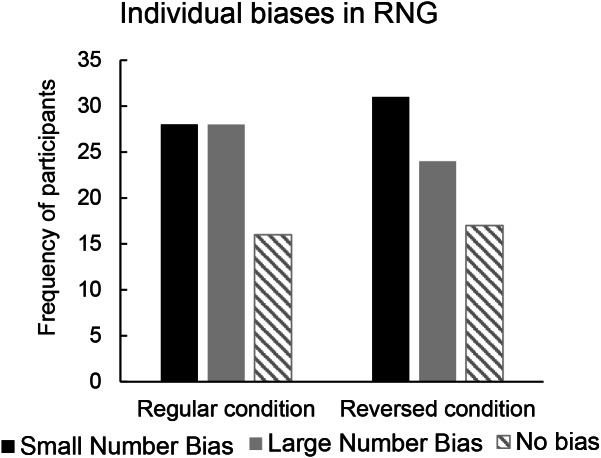
Distribution of participants who generated a higher (“Small Number Bias”), lower (“Large Number Bias”), or equal (“No bias”) frequency of small versus large numbers in both the Regular and Reversed conditions

To better describe results, individual data are plotted in Fig. 4, showing the frequency of participants who generated a higher, lower, or equal frequency of small/large numbers in both the Regular and Reversed conditions. Moreover, Table 3 displays how participants are distributed based on the total amount of small/large numbers they generated in the two conditions.

**Table 3:** Frequency of participants generating varying amounts of small and large numbers across conditions, out of the 60 digits required to be generated. For example, in the regular condition, 8 participants generated a total amount of 28 small numbers (i.e., numbers ranging from 1 to 6), 14 participants generated a total amount of 29 small numbers, and 15 participants generated 30 small numbers

Amount of numbers generated		Frequency of participants per amount of small/large numbers generated														
		23	24	25	26	27	28	29	30	31	32	33	34	35	36	37
Regular condition	Small numbers (1–6)	0	1	2	4	2	8	14	15	11	10	3	2	0	0	1
	Large numbers (7–12)	1	0	0	4	4	9	12	15	15	7	3	2	0	1	0
Reversed condition	Small numbers (1–6)	0	0	0	2	6	8	8	20	11	4	5	7	1	1	0
	Large numbers (7–12)	0	2	2	7	6	5	10	19	7	8	6	1	0	0	0

To summarize, results overall show that some numbers are produced more often than others depending on the condition. In particular, RNG determined a pattern of number production that tends towards the SNB in general, however, this tendency reaches the significance level only in the Reversed condition, where significantly more small numbers than large numbers are produced (Fig. 3); conversely the SNB is disrupted in the Regular condition. It must be noted that in the Reversed condition, the numbers that are produced more often presents at the same time the characteristics of being the smallest and the most leftward. Thus, the SNB emerged only in the condition where the effect of both mechanisms (i.e., frequency of use and pseudoneglect) add up. Finally, the analyses that aimed to determine which account best explains SNB did not provide a clear result. Such comparisons seem to point to frequency of use as the mechanism that plays the biggest role in driving SNB, however a cautious interpretation is needed.

## Discussion

The aim of the present study was to investigate the phenomenon of “Small Number Bias” (SNB; Loetscher & Brugger, 2007), which refers to the tendency of individuals to generate more small numbers than large ones when asked to produce random numbers. Two possible theoretical explanations for this bias are addressed in the present study. According to the first one, referred to as “pseudoneglect” (Jewell & McCourt, 2000), the SNB would derive from a spatial preference where attention is automatically biased towards the left side of the mental number line. According to the second one, referred to as “frequency of use”, the SNB would arise from the fact that smaller numbers are used more frequently than larger ones in daily life (Dehaene & Mehler, 1992).

While the two explanations are not mutually exclusive, it is important to determine to what extent each of them explains this phenomenon, as they underlie different processes. In this experiment, the pseudoneglect and the frequency of use accounts were systematically explored together for the first time. This was achieved by using a common numerical display, i.e., a clockface, whose characteristics allow to observe the contributions of the two mechanisms separately. Participants were exposed to these configurations while performing random number generation. The frequency with which they produced each number was analysed to identify and address any systematic bias in participants’ number production patterns.

In line with other examples of RNG tasks (Loetscher & Brugger, 2007; Loetscher et al., 2008a, b, 2010), this novel version of the task reveals participants’ general tendency to produce small numbers more often than large ones. In particular, this tendency is more pronounced in presence of a reversed clockface display, in which small numbers are located on the left and large ones are on the right. Indeed, a significant SNB emerged in the Reversed condition. Interestingly, the numbers that are produced more often in this condition are not only the smallest but are also the most leftward. This result thus supports both pseudoneglect, which predicted a greater production of leftward (small) numbers in this condition, and frequency of use, which predicted a greater production of small numbers, independently from the spatial arrangement of numbers on the clockface. The joint action produced by the two mechanisms operating in the same direction could thus have determined SNB.

Conversely, when exposed to a regular clockface, in which small numbers are on the right and large ones are on the left, participants did not exhibit SNB. On one hand, the lack of SNB observed in the Regular condition seems to contradict the frequency of use explanation, which predicts SNB independently from the spatial arrangement of numbers. On the other hand, no overproduction of large (leftward) numbers was detected solely because they are located on the left side, either. Hence, results from this condition do not provide clear support either to the pseudoneglect or to the frequency of use account.

The absence of SNB in the regular clockface condition could be attributed to the opposite directions of the two mechanisms operating simultaneously (pseudoneglect and frequency of use). It is hard to determine how individual behaviour was affected by these two factors, however it is quite clear that the SNB in the reversed clockface condition is observed at group level, similar to other numerical cognition effects, such as the SNARC effect. Indeed, although widely documented at the group level (Wood et al., 2008, for a meta-analysis; Toomarian & Hubbard, 2018, for a review), the SNARC effect is considerably variable at the individual level: only a minority of participants show a reliable effect, and approximately half do not exhibit any spatial-numerical association (Cipora et al., 2019; Roth et al., 2024). To summarize, the SNB emerged only in the condition where the effect of both mechanisms (i.e., frequency of use and pseudoneglect) add up, namely in the Reversed condition. This suggests that the influence of both factors is necessary for the bias to manifest.

The additional analyses conducted to determine which theory best explains the SNB produced ambiguous results but highlight an important point for discussion. Specifically, the comparison between the most leftward and most rightward numbers offered no clear indication, further diminishing the likelihood that pseudoneglect influenced performance. However, the comparison between the smallest and largest numbers across conditions revealed a difference that approached significance. This latter finding appears more suggestive of the involvement of frequency of use, which may have played the predominant role in driving the SNB. While this evidence alone may not provide definitive proof, it offers valuable insights that contribute to shed light on the bias.

The presence of SNB in the Reversed condition is not surprising, as the reversed clockface reflects the spatial arrangement that numbers have in the MNL. In this regard, the “MNL-like” configuration presented during RNG might have acted as a “guide” to number production, reinforcing a natural tendency to produce small numbers in a higher amount. This is in line with Loetscher & Brugger (2008) who reported that visual imagery of a ruler (i.e., MNL) enhanced participants preference for small number production in RNG. Differently, the regular clockface is opposite to the MNL, and might interfere with participants’ natural habit of sourcing numbers from the left part of space.

The present study’s findings are partially consistent with previous results from a similar experiment. Mingolo et al. (2024) reported a RNG experiment on numbers ranging from 1 to 12. Participants performed RNG with their eyes closed while mentally visualizing either a ruler (reflecting both MNL and the Reversed condition from the present study) or a regular clockface. Different from the present results, a reliable SNB was observed in *both* conditions. This difference in findings can be attributed to variations in task requirements. When a context is introduced only at the beginning of the experiment and not reinforced throughout it cannot significantly influence SNB, as reported in Mingolo et al., 2024. In contrast, the present study explicitly instructed participants to rely on the clockface configuration to generate numbers during task execution. This explicit instruction likely enhanced the clockface context’s influence, leading to the elimination of SNB in the regular clockface condition.

Notably, our task differs from traditional RNG paradigms in one key aspect: the numbers were visually presented to participants throughout the experiment. This procedure reduces the reliance on working memory, a component typically engaged in standard RNG tasks where participants must internally generate numbers within a fixed range. Nonetheless, the typical pattern of results (i.e., SNB) emerges when the small-left / large-right arrangement of numbers is displayed, similar to the MNL. This procedure allowed us to investigate two theoretical explanations for the SNB, which pertain to different aspects of numerical processing. Pseudoneglect is rooted in a visuospatial mechanism, while frequency of use is more closely associated with a verbal mechanism. The emergence of SNB only in the condition where both pseudoneglect and frequency of use operate in the same direction suggests the simultaneous involvement of visuospatial and verbal processing in number generation.

## Conclusions

The present study explored the theoretical foundations of the Small Number Bias, which reflects an overrepresentation of smaller numbers in the human mind. Two possible explanatory accounts were considered: pseudoneglect, which involves a spatial preference for the left side of the mental number line, and the frequency of use, where smaller numbers are more commonly used in daily life. The study systematically explored both accounts using a clockface display to investigate their separate contributions. Participants generated random numbers while visualizing either a regular or a reversed clockface. Results showed that participants exhibited significant SNB when viewing a reversed clockface (small numbers on the left), supporting both pseudoneglect and frequency of use theories. However, no SNB was observed with a regular clockface (small numbers on the right). This indicates that SNB emerges only when pseudoneglect and frequency of use align, suggesting that both visuospatial and verbal processing contribute to SNB.

## Data Availability

Raw data are available at the following link: https://osf.io/dmtqg/?view_only=606e06dbafc74230a6b068324a636e92.
